# Dutch translation and cross-cultural adaptation of the PROMIS^® ^physical function item bank and cognitive pre-test in Dutch arthritis patients

**DOI:** 10.1186/ar3760

**Published:** 2012-03-05

**Authors:** Martijn AH Oude Voshaar, Peter M ten Klooster, Erik Taal, Eswar Krishnan, Mart AFJ van de Laar

**Affiliations:** 1 Institute for Behavioural Research, University of Twente, Drienerlolaan 5, Enschede, 7522 NB, The Netherlands; 2 Department of Medicine, Stanford University School of Medicine, 1000 Welch Road, Suite 203, Palo Alto, CA 04304, USA; 3 Department of Rheumatology, Medisch Spectrum Twente, Haaksbergerstraat 84, 7513 EA, Enschede, The Netherlands

## Abstract

**Introduction:**

Patient-reported physical function is an established outcome domain in clinical studies in rheumatology. To overcome the limitations of the current generation of questionnaires, the Patient-Reported Outcomes Measurement Information System (PROMIS^®^) project in the USA has developed calibrated item banks for measuring several domains of health status in people with a wide range of chronic diseases. The aim of this study was to translate and cross-culturally adapt the PROMIS physical function item bank to the Dutch language and to pretest it in a sample of patients with arthritis.

**Methods:**

The items of the PROMIS physical function item bank were translated using rigorous forward-backward protocols and the translated version was subsequently cognitively pretested in a sample of Dutch patients with rheumatoid arthritis.

**Results:**

Few issues were encountered in the forward-backward translation. Only 5 of the 124 items to be translated had to be rewritten because of culturally inappropriate content. Subsequent pretesting showed that overall, questions of the Dutch version were understood as they were intended, while only one item required rewriting.

**Conclusions:**

Results suggest that the translated version of the PROMIS physical function item bank is semantically and conceptually equivalent to the original. Future work will be directed at creating a Dutch-Flemish final version of the item bank to be used in research with Dutch speaking populations.

## Introduction

Physical function is impaired by musculoskeletal disease in patients with arthritis. So physical function has a long tradition as a core outcome domain in this field [1,2]. Since its introduction in the 1980s, the Health Assessment Questionnaire-Disability Index (HAQ-DI) has become a standard outcome measure of physical function in clinical trials and observational studies [3]. However, over time some considerable limitations of the scale have become apparent. The most frequently cited of these are its burdensomeness to patients and administrators due to a high number of questions and complex scoring and its relatively short measurement range, which leads to ceiling effects and reduced sensitivity to measure change, especially for relatively high-functioning patients [4-9].

Recent studies indicate that these shortcomings can be overcome by the development of a calibrated item bank, using Item Response Theory (IRT) as a statistical method. From such an item bank, short forms or computerized adaptive testing protocols (CAT) of physical function can be developed [10,11]. Both methods of assessment help ensure that patients only respond to questions that are relevant to their specific level of disability and that only minimal questions need to be answered by patients, while retaining or surpassing the measurement precision of classical instruments [12]. A number of efforts have already demonstrated that IRT-based measurement has the potential to provide more robust and responsive assessment of physical function in arthritis than classical counterparts [13-16].

Perhaps the most ambitious effort to bring in modern testing approaches to measurement of health status is the Patient Reported Outcomes Measurement Information System (PROMIS^®^). This NIH initiative aims to revolutionize the way patient-reported outcome tools are selected and employed in clinical research and practice evaluation by developing item banks and CATs for important health-related quality of life outcome domains [17]. A recent study of patients with RA showed that a 10-item simulated PROMIS^® ^physical function CAT outperformed the legacy HAQ-DI in terms of measurement precision and width [10]. These findings underscore the role that computer-based assessment of physical function with the PROMIS physical function item bank could play in bypassing the trade-off between measurement precision and burdensomeness to patients that necessarily exists when using fixed length tests.

Now that the PROMIS physical function item bank is ready for use and studies have demonstrated its benefits over legacy instruments such as the HAQ-DI, an important next step is to disseminate it to other countries and cultures. It is generally recognized that if measures are to be used across cultures, the items must not only be translated well linguistically, but also must be adapted culturally to maintain the content validity of the instrument at a conceptual level across different cultures [18,19]. To date no cross-cultural adaptations of the PROMIS item bank are available for use in the Netherlands. Moreover, although the psychometric properties of most Dutch translations of frequently used physical function instruments are well known (e.g., the HAQ [16] and AIMS2 [20]) this is one of the first studies to offer a detailed description of the methodology of the cross-cultural translation of physical function items to the Dutch language. In fact, the International Quality of Life Assessment (IQOLA) project is the only such effort in the peer-reviewed literature. The IQOLA project documents the translation of the MOS physical functioning scale using rigorous methodology and showed that physical function items are particularly difficult to translate because they frequently refer to activities not common outside the USA [21,22].

The aim of this study was to cross-culturally translate the PROMIS^® ^physical function item bank items to Dutch, according to strict and rigorous guidelines for the translation of health-related quality of life instruments and to pretest the translated version in a sample of Dutch patients with arthritis.

## Material and methods

### PROMIS^® ^physical function item bank

The PROMIS physical function item bank measures self-reported, current capability of physical activities. The item bank contains 124 questions assessing the functioning of the upper extremities (dexterity), lower extremities (walking or mobility), and central regions (neck and back), as well as instrumental activities of daily living, such as running errands [23]. Questions were derived from 1865 extant physical function items that were identified in a systematic review of the literature on existing physical function instruments. Eligible items underwent extensive qualitative evaluation with patient surveys and focus groups [24]. Subsequently, items were standardized in terms of item stems and response options. The resulting item bank was empirically tested in more than 21,000 persons from the general population, which included clinical samples of 1473 adults with self-reported arthritis [10,14,25].

### Adaptation process

Various guidelines for the process of cross-cultural translation of health-related quality of life instruments have been proposed [12,18,19,26]. There is consensus in the literature that the main elements in the procedure should include: (I) forward translation into the target language; (II) back-translation into the source language by native speakers; (III) consensus meetings of people involved; and (IV) testing of the translation to the target language. What the scope of this final stage should be differs between guidelines, however. Although there is consensus that the main objective of this phase is to verify that all questions are comprehended as intended by respondents, Beaton et al. propose to investigate the distribution of responses as well, to check for high proportions of missing items and single responses [19]. However, for the pretesting of item banks with large numbers of items, respondents are usually debriefed about subsets of items. This makes it a cumbersome process to enroll enough respondents to obtain meaningful information about these issues. Therefore these issues might better be investigated when data is being collected for calibrating the translated item bank.

#### Step 1: Forward translation

Two translators, both bilingual health professionals working in the field of rheumatology with Dutch as their mother tongue and proficient in English independently produced a forward translation of the 124 items. Both translators were provided with a spread sheet containing item definitions and potential item-specific translatability problems as identified by PROMIS^®^.

#### Step 2: Synthesis of the translations

Inconsistencies between the forward translations were reconciled in a consensus meeting that was attended by both translators and a third health professional working in the field of rheumatology who recorded the process of reaching consensus and participated in the process of reconciling versions to create a synthesis version of the translation.

#### Step 3: Back translation into English

The items were translated back into American English by two professional translators, who were both native speakers of English, proficient in Dutch and living in the Netherlands. One of the translators was a British English native speaker and the other translator was an American English native speaker. Both translators were not informed about the concepts underlying the items' content and were not medically schooled or trained. Back translators were instructed to translate the Dutch items into American English and were told that measurement units of the metric system that feature in the Dutch items should, by approximation, be converted back to the corresponding imperial measurement units that are used in the USA. Following back translation, back translators were given access to the original English items to comment on the initial forward translation.

#### Step 4: Expert committee

Following correspondence with both back translators about the adequacy of the original forward translations, an expert meeting was organised to consolidate all the versions. During the expert meeting, all discrepancies between the translations were reviewed and potential cross-cultural issues were discussed. During the meeting final decisions were made to generate a pre-final version of the PROMIS physical function item bank ready for testing by patients. Besides the input from the back translators, the committee consisted of one practicing rheumatologist, one social psychologist working in the field of rheumatology, one methodologist, and both forward translators.

#### Step 5 test of the prefinal version

The aim of this final stage was to check the understanding and interpretation of the translated items in a population of Dutch patients with arthritis and thereby validate the conceptual equivalence between the US and Dutch versions. To be eligible for inclusion patients had to have a physician diagnosis of rheumatoid arthritis, osteoarthritis, or psoriatic arthritis, be at least 18 years old, and be free of any concurrent medical or psychiatric condition that might preclude participation in the study. As it would be overly burdensome for participants to be interviewed about all 124 items, a sampling scheme was applied that allowed for each participant to be interviewed on between 25 and 33 items. This allowed each item to be evaluated by five patients, as stipulated in the PROMIS qualitative item review protocol [27]. Informed consent was provided according to the Declaration of Helsinki and obtained from all participating patients. According to the Dutch law for medical research with humans, approval by an ethical committee was not indicated for this study. Three Step Test Interview (TSTI) method, which is a cognitive interviewing method based on think aloud methodology [28]. The TSTI is a qualitative research instrument specifically designed for testing self-assessment questionnaires that has proven to be effective in identifying reporting errors in health-related quality of life questionnaires [29]. The TSTI consists of the following three steps: respondent driven observation of response behavior; interviewer driven follow up probing aimed at identifying gaps in observational data; and interviewer driven debriefing aimed at eliciting experiences and opinions of respondents.

Content analysis and descriptive summary statistics were used to evaluate the information gathered during the cognitive debriefing interviews and to characterize the participant sample. The analysis was based on notes taken by the interviewer and done on an item-by-item basis. This information was used either as a final seal of approval of an item or as input for item revision.

## Results

### Steps 1 to 3: Translation process

Only minor inconsistencies were observed between both forward translations, mostly related to different word choices. The term 'full pint container' used in item 31 does not have a direct Dutch translation that would be relatable for patients as a concept; therefore it was replaced by the term 'glass containing half a liter of water'. The only inconsistency between translations that required more elaborate discussion concerned the stem of the first set of items (are you able to). From a semantic point of view it can be translated as 'kunt u' (can you) and 'bent u in staat om' (are you able to). Although the former translation is less literal, it conveys the same meaning as the latter translation and it has the added benefit of resulting in less structurally complex sentences, which is why it was eventually adopted.

### Step 4: Expert committee

During the expert committee meeting it was decided that inconsistencies between the back translations and the original items were too minor to warrant changing the initial forward translations. A number of cross-cultural issues were identified, however, that needed to be addressed: First, in the Netherlands street patterns are irregularly shaped, unlike in the US where city blocks are a central element in urban planning, so to walk a block will have a different meaning to different persons. Therefore item 17 was changed to: can you walk 150 meters (approximately 150 yards). Second, doors with door knobs are quite uncommon in the Netherlands. Most doors have latches. To ensure that item 20 would be understandable for all patients we replaced the word 'door knob' with 'door latch' Third, the Dutch word for 'liquid' is used mostly to refer to a specific state of matter, rather than referring to something that can be drunk in everyday life. Therefore we replaced the word 'liquid' with 'water' in item 44. Fourth, both items 56 and 59 refer to reaching into low and high cupboards, respectively. However to 'reach into' is not directly translatable to Dutch, the closest approximation being 'to reach for'. Therefore the wording of these items was changed to: 'to get something from low/high cupboards'. Finally, all imperial measurement units were converted to corresponding units in the metric system and rounded to the nearest 0 or 5. An overview of all adjustments relative to the source items is presented in Table 1.

**Table 1:** Adjustments of expressions and items made during the process of translating the item bank

Source item	Dutch version; English equivalent	Final Dutch version	Reason for adaptation
Are you able to walk a block on flat ground?	Are you able to walk 150 meter on flat ground?	Kunt u 150 meter lopen op vlakke ondergrond?	'Block' is a poorly understood concept in Dutch.
Are you able to push open a door after turning the knob?	Are you able to push open a door after pushing down the latch?	Kunt u een deur open duwen na nadat u de klink naar beneden heeft geduwd?	Doors in the Netherlands do not have knob-type door operating mechanisms.
Are you able to pour liquid from a bottle into a glass?	Are you able to pour water from a bottle into a glass?	Kunt u water vanuit een fles in een glas schenken?	The word 'liquid' is mostly used to refer to a specific state of matter, rather than something that can be drunk.
Are you able to reach into a high cupboard?	Are you able to retrieve something from a high cupboard?	Kunt u iets uit een hoog keukenkastje pakken?	To reach into is not directly translatable to Dutch.
Are you able to reach into a low cupboard?	Are you able to retrieve something from a low cupboard?	Kunt u iets uit een laag keukenkastje pakken?	To reach into is not directly translatable to Dutch.

### Step 5: Pretest

The resulting item pool was pretested in 20 patients with rheumatic diseases. Some clinical and demographic information about the participating patients is listed in Table 2.

**Table 2:** Patient characteristics (n = 20)

Characteristic	N (SD)	Mean range
Diagnosis (%)		
Rheumatoid arthritis	15	
Osteoarthritis	3	
Psoriatic arthritis	2	
Gender (% female)	70%	
Age	53.7 (7.5)	39-63
Disease duration in years		
Mean	10.6 (7.9)	3-31

Interview data indicated that questions were well understood by patients. Generally, the questions were filled in quickly at a consistent speed. Patients rarely hesitated or corrected a previously reported answer. When thinking aloud, patients tended to reflect on the activities as they carry them out in their daily lives and mentally went over problems that are associated with carrying out the activities. This provided a good indication that patients understood the items as they were intended. The only exception to this concerned item 31 (Are you able to lift one pound (a full pint container) to shoulder level without bending your elbow?). All five patients took considerably more time in answering this question and four out of five patients asked for additional clarification regarding the meaning of the question. These observations indicate that this item was not well understood by patients and the formulation was changed. In addition, two questions with double-barreled content were identified that prohibited respondents from giving a consistent answer. The first was item 52 (are you able to use your hands, such as for turning faucets, using kitchen gadgets, or sewing?). Four out of five patients indicated that it was not possible for them to give a consistent answer to this question. Patients indicated that sewing is an activity much more related to dexterity of the fingers than the other activities, which seem to be more related to gross motor skills and movements of the wrist. The second problem concerned item 122 (are you currently restricted by your health from taking part in physically active sports such as swimming, tennis, or basketball?). Three out of five patients indicated that they experience significantly less difficulty with swimming than with performing either of the ball sports, because swimming is far less strenuous to the joints. Although the activities in these questions serve an illustrative purpose, patients interpreted the question as if they are being asked if they can perform those exact activities. Consequently, it becomes impossible to give a consistent answer in case they experience the activities to be unequally difficult. Because this problem does not appear to stem from the translation of the items or from cross-cultural issues, these items were not changed.

Aside from theses item specific problems, two issues related to the questions in general were identified. The first is that patients often indicated that they missed a reference to time in the questions. The second, related, problem that was encountered with items referring to strenuous activities such as walking or running more than a mile or working in the garden is that patients indicate they can, in principle, perform the tasks referred to in the items but are nevertheless reluctant to do so because they know that they will suffer increased fatigue, stiffness and pain in the days after. As with the double barreled questions, however, these issues do not seem to stem from the translation process and consequently no remedial action was undertaken.

## Discussion

This study describes the process of cross-culturally adapting the PROMIS^® ^physical function item bank items according to rigorous methodological standards for use in the Dutch culture. The main aim of the study was to verify the conceptual equivalence and linguistic validity of the PROMIS physical function items for use in Dutch arthritic patient by using cognitive interviewing methodology.

We noted few inconsistencies between both forward translations and between the back translations and the original items, indicating that the translated version is reflecting the same item content as the original version. In fact, the back translation from the American native back translator was almost perfectly equivalent to the original items that did not require cross-cultural adaptation. An explanation for this might be that all questions in the PROMIS physical function item bank refer to very concrete, every day activities. Moreover, the items in the original American item bank already underwent rigorous qualitative assessment by experts and respondents to ensure that the reading level would be suitable for all respondents [24]. Most changes that were made in the adaptation process concerned cross-cultural issues. In all cases these changes concerned substituting uncommon or non-used concepts and objects for objects and concepts that are better suited to the Dutch culture. For most questions it was possible to make small conceptual changes while retaining the difficulty level of questions. For instance, imperial measurement units were converted to grossly corresponding units of the metric system. However, some concepts such as walking a block do not have a straightforward equivalent translation in Dutch, as was noted in the IQOLA project as well [22]. Sometimes more radical changes were required. For example, opening a door with a door knob may be more difficult to do than opening a door with a door latch, especially for patients with arthritis. This may undermine the cross-cultural measurement equivalence of this specific item. However, the impact this might have on the item's function needs to be investigated empirically. The HAQ [30] and SF-36 [22] physical functioning items have previously been translated to the Dutch language and also feature in adapted form (i.e. more response options and sometimes changed wording) in the PROMIS physical function item bank. Our Dutch translation of the 15 HAQ items that were left unchanged by PROMIS were equivalent to the items in the Dutch Consensus HAQ, except for item 10: 'are you able to wash and dry your body?'. We chose a literal translation whereas in the Dutch HAQ the literal English translation would read: 'Are you able to wash and dry your body yourself?'. Also the sequence of words of item 16: 'Are you able to open previously opened jars?' is slightly different in our translation than in the Dutch HAQ. In the Dutch translation of the SF-36 items, we deviated from the Wagner et al. translation for 2 out of 10 items; We translated mile in the item: 'Does your health now limit you in walking more than a mile?' as 1.5 kilometres instead of 1, in order to assure the measurement equivalence of the item, cross-culturally. Also Wagner et al translated 'bowling' and 'playing golf' as 'swimming' and 'cycling', respectively in the item: 'Does your health now limit you in doing moderate activities, such as moving a table, pushing a vacuum cleaner, bowling, or playing golf?', whereas we chose to preserve the original content for this item.

There is general consensus in the literature that the process of translating a questionnaire or item bank should be followed by a pretest to assess the success of the translation by verifying that the item wording is clear, unambiguous, and permits respondents to successfully answer the questions [18,19,26]. Cognitive interviewing techniques are well suited for this because the verbalized reflections of patients provide an excellent source of information to verify that questions are understood and answered to as intended. Two major types of cognitive interviewing methods are generally distinguished in the literature: think-aloud interviewing and verbal probing techniques [31]. The item review of the original PROMIS^® ^item banks employed verbal probing techniques. In this approach respondents undergo a structured interview where the goal is to use targeted probing to guide the interchange in a way that is controlled mainly by the interviewer. The main advantage of this approach is that the interviewer can focus on particular areas that appear to be relevant as potential sources of response error. Item review of the Dutch translation of the PROMIS physical function item bank, however, employed think-aloud methodology. The main advantage of think-aloud methods is that there is minimal interviewer-imposed bias, and, consequently, unanticipated problems in the response behavior of participants are more likely to be detected. These two methods therefore complement each other: Verbal probing techniques provide a good way to verify that questions are comprehended as intended on a semantic and conceptual level in a structured setting where the interviewer controls the course of the exchange. However response errors can occur even though questions are correctly understood by patients if questions have different meaning to patients than initially expected by the developers of the questions. For instance, in this study it was revealed that patients consistently had problems with item 121 (are you currently restricted by your health from taking part in physically active sports such as swimming, tennis or basketball?), because they perceive these activities to be unequally difficult. This type of important issue that precludes patients to give a consistent answer is more likely to be detected if patients are allowed to freely verbalize their thoughts when formulating an answer to the question.

### Future work

Given the objective of PROMIS to develop one version for multiple countries instead of country-specific versions of the same language, the translation effort described in this study is currently incorporated in the official Dutch-Flemish PROMIS item bank translation process, together with three independent translations produced by FACIT. Following the PROMIS methodology, the most appropriate translation considering both the Dutch and Flemish cultures will be chosen for each item. The consensus translation will then be tested through cognitive debriefing in a small sample of members from the general population in both the Netherlands and Belgium to produce a definite official translation. After that phase, data will be collected to calibrate the Dutch translation of the PROMIS physical function item bank and to assess its psychometric performance in several chronic diseases, including patients with arthritis.

## Conclusions

This study describes the process of cross-culturally adapting the PROMIS^® ^physical function item bank for use with Dutch patients with arthritis. This is, to our best knowledge, the first study describing the cross-cultural adaptation of any of the PROMIS^® ^item banks. The rigorous translation methodology employed ensures that the Dutch version is semantically and conceptually equivalent to the original. Furthermore item review verified that all Dutch items are comprehended by patients as they were intended although some minor general problems in the response process persist that are most likely to be prevalent in the original version of the item bank as well.

## Abbreviations

CAT: computerized adaptive testing; HAQ-DI: Health Assessment Questionnaire-Disability Index; IRT: Item response theory; IQOLA: International Quality of Life Assessment; PROMIS: Patient-Reported Outcomes Measurement Information System; TSTI: Three step test interview.

## Competing interests

The authors declare that they have no competing interests.

## Authors' contributions

MOV helped conceive the study design, carried out data collection, conducted the initial analyses, wrote the first draft of the manuscript and had primary responsibility for the manuscript process. PTK, ET and MVDL supervised the study and the interpretation of the results. EK helped conceive and supervised the study design. All authors critically reviewed, contributed and approved the final manuscript.
